# Soil aggregate and microbial traits mediate soil organic carbon accumulation in a paddy field under long-term elevated CO_2_ and warming

**DOI:** 10.1093/ismeco/ycag206

**Published:** 2026-07-14

**Authors:** Yuan Liu, Mengting Li, Jiayi Xiong, Feng Li, Cheng Liu, Xiaoyu Liu

**Affiliations:** Anhui Province Key Laboratory of Pollutant Sensitive Materials and Environmental Remediation, College of Life Science, Huaibei Normal University, Huaibei 235000, Anhui, China; Anhui Province Key Laboratory of Pollutant Sensitive Materials and Environmental Remediation, College of Life Science, Huaibei Normal University, Huaibei 235000, Anhui, China; Anhui Province Key Laboratory of Pollutant Sensitive Materials and Environmental Remediation, College of Life Science, Huaibei Normal University, Huaibei 235000, Anhui, China; Anhui Province Key Laboratory of Pollutant Sensitive Materials and Environmental Remediation, College of Life Science, Huaibei Normal University, Huaibei 235000, Anhui, China; Institute of Eco-environmental Research, Zhejiang University of Science and Technology, Hangzhou 310023, China; Institute of Resources Ecosystem and Environment of Agriculture, Nanjing Agricultural University, 1 Weigang, Nanjing 210095, China

**Keywords:** elevated CO_2,_ rice paddy, microbial necromass, microbial community, soil aggregate

## Abstract

Climate change is increasingly impacting ecosystem functions and soil microorganisms, altering the accumulation and stability of soil organic carbon (SOC). However, the effect of climate change on SOC dynamics at the aggregate scale is poorly understood. Here, we conducted a 13-year experiment to investigate how elevated CO_2_ (+200 ppm) and warming (+2°C) affect microbial community and necromass, and potential enzyme activities within soil aggregates in a paddy field. Results showed that long-term elevated CO_2_ and warming significantly increased SOC and total nitrogen contents but reduced soil pH and total phosphorus (TP) within all three aggregate fractions. The SOC and TP contents were the most closely related to changes in microbial communities in aggregates. Elevated CO_2_ and warming significantly increased microbial activities, specially increasing both fungal and bacterial abundances, leading to increases in C- and N-potential enzyme activities in large macroaggregate and microaggregates. In contrast, P-acquiring enzyme activities and the stoichiometry for P:N enzyme ratio significantly decreased with elevated CO_2_ and warming. Furthermore, climate change effects on the microbial necromass varied across aggregate sizes. Elevated CO_2_ significantly increased fungal necromass C and its contribution to SOC in large macroaggregates, while warming increased bacterial necromass C and its contribution to SOC in microaggregates. The SOC was positively correlated with microbial necromass and fungal necromass C in macroaggregates. The highest microbial necromass C in large macroaggregates may be attributed to higher fungal abundance and lower microbial enzyme activities. These results suggest that climate change may regulate SOC dynamics by affecting microbial growth and activity in soil aggregates. Our study highlights that soil aggregates stratify microbial communities and necromass, which improve our understanding the responses of agricultural ecosystem C cycling to future climate change.

## Graphical Abstract

**Figure:**
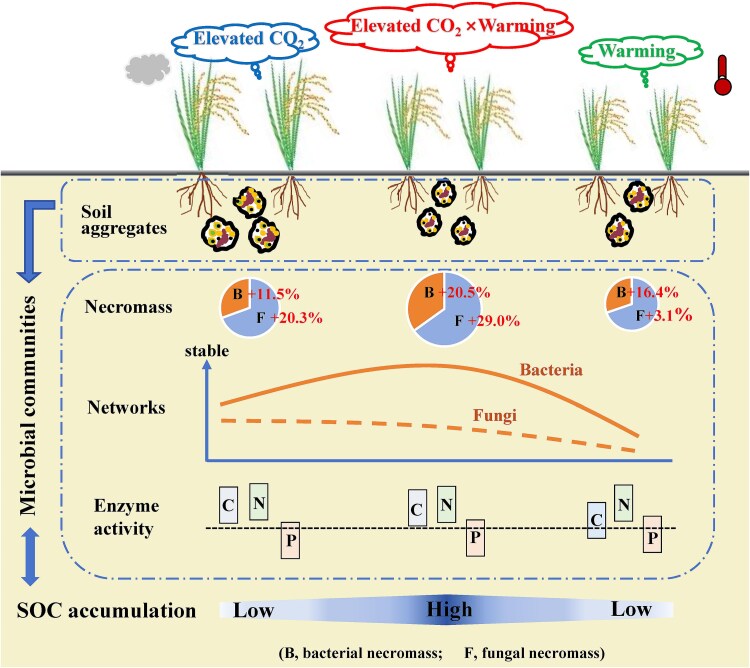

## Introduction

Climate change is reshaping terrestrial carbon (C) storage and ecosystem functions, driven by rising atmospheric CO_2_ concentrations and surface temperatures [1, 2]. As the largest terrestrial C pool (~1570 Pg), increasing soil organic carbon (SOC) sequestration is crucial for sustaining soil fertility and mitigating climate change [3]. In recent years, the stability of SOC pool was fundamentally regulated by microbial growth, reproduction, and death [4, 5]. Thus, understanding the mechanisms of microbial-derived C to climate change may improve the prediction of ecosystem SOC pool to future climate change conditions.

As the main features of climate change, elevated CO_2_ and warming can affect the formation and accumulation of microbial-derived C directly or indirectly by altering soil properties, plant traits, microbial community, and enzyme features [6]. Elevated CO_2_ often enriches microbial diversity in paddies by stimulating root exudation and labile C availability and accelerated SOC turnover via the priming effect [7, 8]. Warming can influence the accumulation of microbial-derived C by stimulating microbial anabolism and altering substrate availability, plant residue quantity and quality [9, 10]. Although the microbial responses to elevated CO_2_ and warming were well known, microbial-derived C response to elevate CO_2_ and warming were highly variable, depending on different microbial necromass C (fungal and bacterial necromass) and microbial traits [11, 12]. Global analysis indicated that microbial necromass contributes an average of 51% to SOC in croplands [13]. The relationship between microbial necromass and microbial community underscores the necessity to understand the formation and stability of SOC under climate change scenarios.

As the basic units of soil structure, soil aggregates regulate the SOC turnover and may influence microbial community composition and activity [14]. The turnover of SOC and aggregates is a dynamic process, and the SOC dynamics are closely related with soil aggregates by providing habitats for microbial access to SOC and physically protected SOC storage [15]. Fungal groups were found to occupy macroaggregates which contain relatively active and labile C from plant residue and mycelium, whereas bacteria often occupy microaggregates to evade predators. Thus, the turnover rate of SOC increases with larger aggregate sizes [16, 17]. The formation and stabilization of aggregates and SOC are also influenced by microbial traits, which change under elevated CO_2_ and warming [18, 19]. Climate change has been shown to affect microbial necromass and activity differentially among different aggregate fractions. Elevated CO_2_ increased fungal SOC turnover in large macroaggregates through enhanced plant photosynthesis and root-derived C with increasing aggregate size [20, 21]. Likewise, warming was shown to accelerate microbial decomposition in macroaggregates, and enhancing microbial necromass through increased plant inputs [22–24]. Therefore, SOC dynamics and microbial activity under elevated CO_2_ and warming depends on soil aggregate fraction, with potential implications for microbe-mediated C cycling feedbacks to climate change [25–27]. However, the relationships among SOC accumulation, microbial traits, and edaphic factors under climate change at aggregate scales is very limited, especially for combined effects of elevated CO_2_ and warming [28].

Here, we conducted an ongoing 13-year field experiment to examine how elevated CO_2_ and warming affect microbial responses in different aggregate sizes and its potential impact on SOC accumulation in a flooded paddy. The objectives of the study were to: (i) quantify changes in soil aggregation, aggregate-associated C, and microbial necromass under elevated CO_2_, warming, and their combination; (ii) evaluate microbial composition and functions within aggregate size fractions, their influence on nutrient cycling, and their overall impact on soil C pools. We hypothesized that elevated CO_2_ and warming would alter microbial compositions and increase specific enzyme activities. These microbial responses and necromass accumulation (bacterial and fungal necromass) are expected to differ across different aggregate fractions, potentially altering the SOC accumulation and stabilization.

## Materials and methods

### Experimental site and design

The open-air field experiment was established in 2010 at the Institute of Resource, Ecosystem, and Environment of Agriculture (IREEA) in Changshu City, China (31°30′N, 120°33′E), within a subtropical monsoon climate zone (mean annual temperature: 16°C; precipitation: 1150 mm). The site features a winter wheat (*Triticum aestivum* L.) and summer rice (*Oryza sativa* L.) rotation on gleyic stagnic anthrosol soil (clay loam texture: 33.8% sand, 38.6% silt, 27.6% clay). Topsoil properties included pH 7.0, SOC 16.2 g kg^−1^, total nitrogen (TN) 1.9 g kg^−1^, and bulk density 1.2 g cm^−3^ [7, 29].

**Figure 1:**
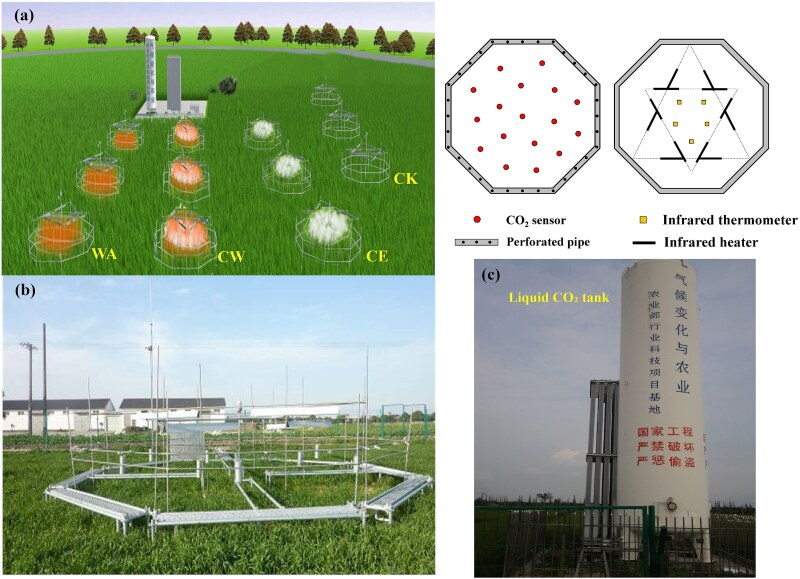
The details of the simulated climate change experiment in this study. Schematic diagram of the free-air CO_2_ enrichment and infrared heating facility with 12 50 m^2^ octagonal rings (a). Pictures of the actual set-up (b) and liquid CO_2_ tank (c). CK, ambient control; CE, CO_2_ enrichment; WA, canopy warming; CW, CO_2_ enrichment plus canopy warming.

The field experiment consisted of two levels of CO_2_ concentration and temperature and four treatments included ambient CO_2_ and temperature (CK), elevated CO_2_ (CE: 550 ppm), canopy warming (WA: +2°C), and combined CO_2_ + warming (CW). Treatment was conducted in triplicate, and each plot was applied to octagonal plots (8 m diameter, 50 m^2^ area) buffered by 28 m open fields to minimize cross-contamination (Fig. 1). The elevated CO_2_ level and canopy temperature were maintained throughout the day during the growing season. Pure CO_2_ (99.99%) was dispensed via eight perforated pipes, regulated by seventeen Li-COR sensors and automated pumps to maintain 550 ppm. The speed of CO_2_ release was manipulated automatically by wind direction and velocity. Total of 12 infrared heaters (2000 W; Kalglo Electronics) were mounted in two triangular configurations and positioned 1.2 m above plants in each ring. The canopy air temperature was monitored by five infrared thermometers (Apogee SI-121) to maintain +2°C [30, 31].

### Rice cultivation and sample collection

Rice (*O. sativa* L. cv. Changyou No.5) was transplanted in June at 26 hills m^−2^ (three seedlings hill^−1^) and then harvested in November each year. Urea (46% N) was applied at a rate of 188 kg ha^−1^ as basal fertilizer and 150 kg ha^−1^ as topdressing fertilizer Compound fertilizer (N:P_2_O_5_:K_2_O, 15%:15%:15%) was applied at 375 kg ha^−1^ at the heading stage. Irrigation control, pest application, and weed management were followed local practices. Wheat and rice residues were removed from the field after the harvesting. Soil samples were collected before the rice harvest from 2020 to 2023 (27 October 2020, 6 November 2021, and 10 November 2023). Five soil cores (5 cm diameter, 0–20 cm depth) per plot were pooled, stored in rigid containers (4°C), and sieved (<8 mm) to preserve aggregates.

### Soil aggregate fractionation and soil property

Soil aggregates were isolated via wet-sieving [32] to preserve microbial integrity, avoiding air-drying artifacts. Fresh soil (300 g) was gently sieved (30 oscillations/min, 10 min) into three fractions: large macroaggregates (>2 mm; LMA), small macroaggregates (0.25–2 mm; SMA), and microaggregates (<0.25 mm; MI). Each fraction was split: one subsample was stored at 4°C for microbial/enzyme assays; the other was air-dried for physicochemical analyses. Aggregate stability indices (mean weight diameter [MWD], geometric mean diameter [GMD]) were calculated to quantify structural resilience.

Soil pH was measured potentiometrically (1:2.5 soil: water; Mettler-Toledo). SOC was quantified via wet oxidation–titration, TN by semi-micro Kjeldahl digestion, and total phosphorus (TP) via molybdenum-blue spectrophotometry (UV3200) [33].

### Soil amino sugars

Soil amino sugars were determined by gas chromatography (Agilent 7890A, USA). Total amino sugars were calculated as the sum of muramic acid (MurA), glucosamine (GluN) and galactosamine (GalN), and mannosamine (ManN). Microbial necromass C was calculated as follows [27]:


(1) \begin{equation*} \mathrm{Bacterial}\ \mathrm{necromass}\ \mathrm{C}\ \left(\mathrm{BNC}\right)=\mathrm{MurA}\times 45\ 31.3 \end{equation*}

(2) \begin{eqnarray*} && \mathrm{Fungal}\ \mathrm{necromass}\ \mathrm{C}\ \left(\mathrm{FNC}\right)=\left(\mathrm{GluN}/179.2\hbox{--} 2\times \mathrm{MurN}/251.2\right)\nonumber\\&& \qquad \times 179.2\times 10.8 \end{eqnarray*}

(3) \begin{equation*} \mathrm{Microbial}\ \mathrm{necromass}\ \mathrm{C}\ \left(\mathrm{MNC}\right)=\mathrm{BNC}+\mathrm{FNC} \end{equation*}

where 179.2 and 251.2 are the molecular weights of GluN and MurN, respectively. The mean translation values from MurN to BNC are 31.3 and GluN to FNC are 10.8.

### Soil enzyme activities

Six enzymes were assayed to assess nutrient cycling: β-glucosidase (BG), cellobiohydrolase (CB), and β-xylosidase (XYL) for carbon (C) acquisition; N-acetylglucosaminidase (NAG) and leucine aminopeptidase (LAP) for nitrogen (N); and neutral phosphatase (PHOS) for phosphorus (P) [34]. Activities were normalized as ln-transformed sums (C: ln[BG + CB + XYL]; N: ln[NAG+LAP]; P: ln[PHOS]). Enzyme stoichiometry (C:N, C:P, N:P ratios) was calculated to infer microbial nutrient demand.

### Soil DNA extraction and real-time quantitative polymerase chain reaction

Total DNA was extracted (PowerSoil™ Kit; MoBio) from 0.5 g soil, with purity verified via Nanodrop (Thermo Scientific). Bacterial 16S rRNA (338F/518R) and fungal ITS (ITS1F/ITS2R) genes were quantified via qPCR (LightCycler 480; Roche) using tenfold plasmid dilutions (10^−1^–10^−7^) for standard curves. Specificity was confirmed via melt–curve analysis.

### Microbial community composition, co-occurrence network, and functional prediction

The composition of the soil microbial community was assessed via Illumina MiSeq sequencing. The purified amplicons of the bacterial 16S rRNA gene (515F/907R) and the fungal internal transcribed spacer gene (ITS1F/ITS2R) were sequenced on the Illumina MiSeq 2500 platform (San Diego, CA, USA) from Genesky Biotechnologies Corporation (Shanghai, China). Raw sequences were trimmed to remove primers, adaptors, and chimeras using Quantitative Insights into Microbial Ecology (QIIME 1. 9.1 pipeline [35]. The DADA2 plugin was used for quality control and to identify amplicon sequence variants (ASVs). The reads of all samples were normalized to the same sequencing depth to correct for sampling effects. Representative sequences of each ASV were annotated based on the SILVA 138 and UNITE 8.0 databases for bacteria and fungi, respectively [36]. All sequence data in this study have been deposited in NCBI Sequence Read Archive under BioProject PRJNA1444537 and PRJNA1444533 for bacteria and fungi, respectively.

Co-occurrence network analysis was performed to reflect the potential relationships among taxa. The microbial network was investigated with a statistically robust Spearman’s correlation threshold >0.6 and a *P* < .01. Topological features of the co-occurrence network (e.g. nodes, edges, average degree, average path length, linkage density) were calculated using the package igraph in R. The co-occurrence network and topological features were investigated using the psych and igraph packages [37] and visualized using the Gephi 0.10.1 software.

### Data analysis

Statistical analyses were conducted using SPSS 22.0 and the R software v. 4.4.2. Before analysis, all data were checked for normality using Shapiro–Wilk tests and for homogeneity of variance using Levene tests. Two-way analysis of variance (ANOVA) followed by Duncan’s test (*P* < .05) was performed to assess the effects of climatic treatments, aggregates, and their interactions on soil and microbial properties. Principal coordinate analysis (PCoA) based on the Bray–Curtis distance was employed to demonstrate the changes in microbial community structure among climate change treatments and aggregate fractions in R, using the Adonis function. Permutational multivariate analysis of variance (PERMANOVA) with the Adonis function was used to assess the significant differences in microbial communities between different groups. Spearman’s correlation analysis determined the relationships among soil properties, enzyme activities, and microbial parameters. Mantel test analysis determined the relationships between microbial communities and soil environmental factors, enzyme activities, and functional genes in soil aggregates in R with Spearman’s coefficients. Linear mixed model analysis was performed to assess the primary effects of aggregates (A) and climate change treatments (T) on soil and microbial parameters, treating A and T as fixed effects and block as a random effect. The significance of the effects of the model was calculated by assessing the denominator degree of freedom, the *F* and *P*-values from the “nlme” package. Linear regression models were used to assess the relatively important variables for soil nutrient accumulation, employing the “relaimpo” package in R. Partial least squares path model (PLS-PM) was performed to quantify the direct and indirect effects of multiple variables on SOC. The model was constructed based on hypothesized causal relationships with the “plspm” package.

## Results

### Soil properties and microbial abundance in soil aggregates

Overall, elevated CO_2_ increased the proportion of macroaggregates compared to the control, and the aggregate stability (GMD and MWD) was significantly increased under elevated CO_2_ (Table 1). There were significant differences in soil properties and microbial abundance among the climate change treatments and aggregates (Tables S1–S3). Elevated CO_2_ and warming significantly increased SOC and TN with the highest values when elevated CO_2_ combined with warming; however, soil pH and TP in treatments were significantly decreased. The SOC and TN were 17.3%–38.8% and 6.2%–17.1% higher in microaggregates than in the macroaggregates, respectively. Moreover, elevated CO_2_ significantly increased the abundances of bacteria and fungi in each aggregate fraction. A significant interactive effect on fungal abundance was observed between climate change treatments and aggregates (*P* < .05, Table. S4). Elevated CO_2_ significantly increased the fungal abundance, more pronounced in large macroaggregates.

**Table 1:** Soil aggregate fractions and stability under simulated climate change.

Year	Treatment	Soil aggregate fraction (%)			Aggregate stability index	
		LMA (>2 mm)	SMA (0.25–2 mm)	MI (<0.25 mm)	GMD/mm	MWD/mm
2020	CK	14.80%(b)	36.27%(a)	48.93%(a)	0.50 ± 0.05 (b)	1.01 ± 0.08 (b)
	CE	27.30%(a)	37.33%(a)	35.37%(b)	0.76 ± 0.07 (a)	1.43 ± 0.14 (a)
	WA	20.27%(ab)	34.47%(a)	45.26%(ab)	0.57 ± 0.08 (b)	1.17 ± 0.17 (ab)
	CW	29.67%(a)	34.30%(a)	36.03%(b)	0.77 ± 0.11 (a)	1.48 ± 0.14 (a)
2021	CK	18.06%(b)	32.45%(a)	49.49%(a)	0.51 ± 0.06 (b)	1.07 ± 0.06 (b)
	CE	25.45%(ab)	35.69%(a)	38.86%(ab)	0.69 ± 0.07 (ab)	1.35 ± 0.13 (ab)
	WA	21.65%(b)	29.50%(a)	48.85%(ab)	0.55 ± 0.09 (b)	1.16 ± 0.14 (b)
	CW	32.62%(a)	30.61%(a)	36.77%(b)	0.78 ± 0.09 (a)	1.54 ± 0.14 (a)
2023	CK	15.32%(b)	34.44%(a)	50.24%(a)	0.49 ± 0.05 (c)	1.00 ± 0.07 (c)
	CE	23.82%(b)	37.02%(a)	39.16%(b)	0.67 ± 0.05 (ab)	1.31 ± 0.02 (ab)
	WA	20.96%(b)	32.27%(a)	46.77%(a)	0.56 ± 0.06 (bc)	1.17 ± 0.15 (bc)
	CW	31.65%(a)	31.50%(a)	36.85%(b)	0.77 ± 0.07 (a)	1.52 ± 0.11 (a)

CK, ambient control; CE, CO_2_ enrichment; WA, canopy warming; CW, CO_2_ enrichment plus canopy warming; LMA, large macro-aggregates (>2 mm); SMA, small macro-aggregates (0.25–2 mm); MI, micro-aggregates (<0.25 mm). Different letters indicate statistical differences (*P* < .05) among different treatments in each aggregate size.

### Microbial necromass C in soil aggregates

**Figure 2:**
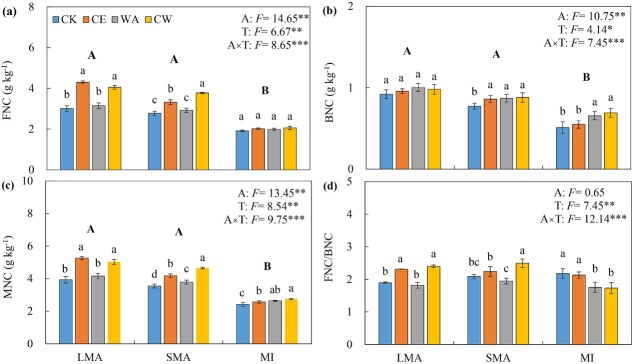
Microbial necromass C in soil aggregates under climate change. Fungal necromass C (FBC, a), bacterial necromass C (BNC, b), microbial necromass C (MNC, c), and the ratio of FBC to BNC (d) are shown. CK, ambient control; CE, CO_2_ enrichment; WA, canopy warming; CW, CO_2_ enrichment plus canopy warming. LMA, large macro-aggregates (>2 mm); SMA, small macro-aggregates (0.25–2 mm); MI, micro-aggregates (<0.25 mm). Letters indicate statistical differences among treatments (lowercase letters) and soil aggregate fractions (uppercase letters). The values are the results of two-way ANOVA analyses on the effects of aggregate fractions (A), climate change treatments (T), and their interactions (A × T). The asterisks “^***^,” “^**^,” and “^*^” indicate significant levels with at *P* < .001, *P* < .01, and *P* < .05, respectively.

Microbial necromass C concentration was significantly affected by climate change treatments and aggregates (Fig. 2). The contents of FNC, BNC, and MNC in large macroaggregates were, on average, 56.8%–89.7% higher than those in microaggregates (Fig. 2a–c). Elevated CO_2_ and warming significantly increased microbial necromass C in each aggregate fraction. Elevated CO_2_ significantly increased microbial necromass C (especially fungal necromass) in large and small macroaggregates, while warming increased microbial necromass C (especially bacterial necromass) in microaggregates. The ratio of fungal to bacterial necromass in large and small macroaggregates were significantly increased with elevated CO_2_, whereas warming decreased the ratio in microaggregates (Fig. 2d). Elevated CO_2_ significantly increased the contribution of fungal necromass C in SOC only in large macroaggregates, while bacterial necromass C under warming made a greater contribution to SOC in microaggregates (Fig. S1). This may mean that the SOC increase in large macroaggregates and microaggregates was mainly due to fungal- and bacterial-derived C, respectively.

### Microbial communities, networks, and potential functions in soil aggregates

**Figure 3:**
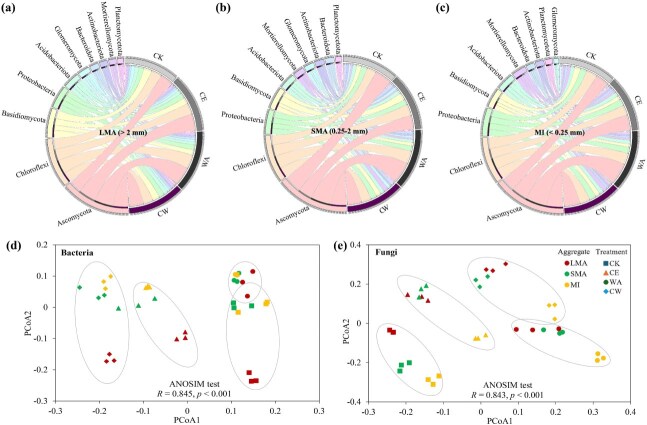
Soil microbial composition and community structure in soil aggregates under climate change. The compositions of the bacterial and fungal communities in LMA (a), SMA (b), and MI (c) are shown using the chord diagram. Principal coordinate analysis (PCoA) was conducted to show the structure of bacteria (d) and fungi (e). CK, ambient control; CE, CO_2_ enrichment; WA, canopy warming; CW, CO_2_ enrichment plus canopy warming. LMA, large macro-aggregates (>2 mm); SMA, small macro-aggregates (0.25–2 mm); MI, micro-aggregates (<0.25 mm).

**Figure 4:**
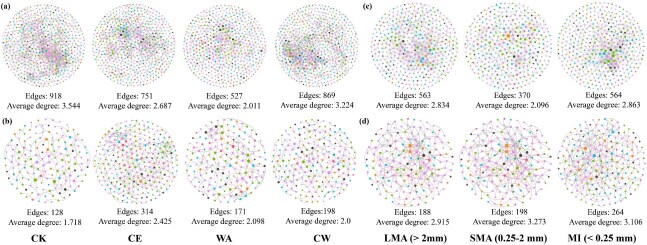
Microbial co-occurrence networks in soil aggregates under climate change. Bacterial co-occurrence networks in treatments (a) and soil aggregate fractions (c); fungal co-occurrence networks in treatments (b); and soil aggregate fractions (d). Edges and average degrees were used as the topological properties of co-occurrence networks.

A total of 2 334 380 and 2 582 044 quality-filtered bacterial and fungal sequences were observed, respectively (Table S5). The Good’s coverage in each sample ranged from 99.84% to 99.98% for bacteria and 99.95% to 99.98% for fungi, respectively, indicating that the sequencing intensity was sufficient to detect the microbial communities. Elevated CO_2_ significantly increased bacterial and fungal α-diversity and OTU richness in microaggregates and large macroaggregates, but decreased those in small macroaggregates (Table S6). Warming significantly decreased the fungal Shannon index across all aggregate fractions. Dominant bacterial phyla included Chloroflexi, Proteobacteria, Acidobacteria, Bacteroidota, and Actinobacteria (Fig. 3). Elevated CO_2_ increased the relative abundance of Acidobacteria (13.4%) and Actinobacteria (8.3%) but decreased Chloroflexi (9.3%) (*P* < .05, Table S7). Warming increased Chloroflexi (6.5%) and decreased Acidobacteria (5.2%). Dominant fungal phyla were Ascomycota (36.7%–62.9%) and Basidiomycota (9.1%–23.5%). Elevated CO_2_ decreased Ascomycota (11.8%) and increased Basidiomycota, with the opposite trend observed under warming. Mortierellomycota increased under both treatments (35.2% and 61.7%, respectively, *P* < .05, Table S7). Climate change treatments and aggregates affected bacterial and fungal community structure (*P* < .05, Fig. 3), with warming specifically impacting fungal community structure. Dominant phyla varied across aggregate fractions and treatments (Fig. S2). Elevated CO_2_ and warming decreased bacterial network complexity (fewer edges, nodes, and average degree), while increasing fungal network complexity (Fig. 4, Table S8).

FAPROTAX analysis revealed that elevated CO_2_ increased the abundance of potentially phototrophs and denitrifiers (*P* < .05, Fig. S3a), while warming increased the abundance of potentially sulfide oxidizers, primarily in microaggregates and decreased methylotrophs. FUNGuild analysis showed that warming increased saprotrophs and decreased arbuscular mycorrhizal fungi (*P* < .05, Fig. S3b). Elevated CO_2_ increased potentially fungal pathogens in macroaggregates and pathogenic bacteria across all aggregate sizes (Fig. S4). According to the prediction of KEGG pathway, the climate change had no impact on bacterial metabolic pathways (Fig. S5).

### Soil enzyme activity and stoichiometry in soil aggregates

**Figure 5:**
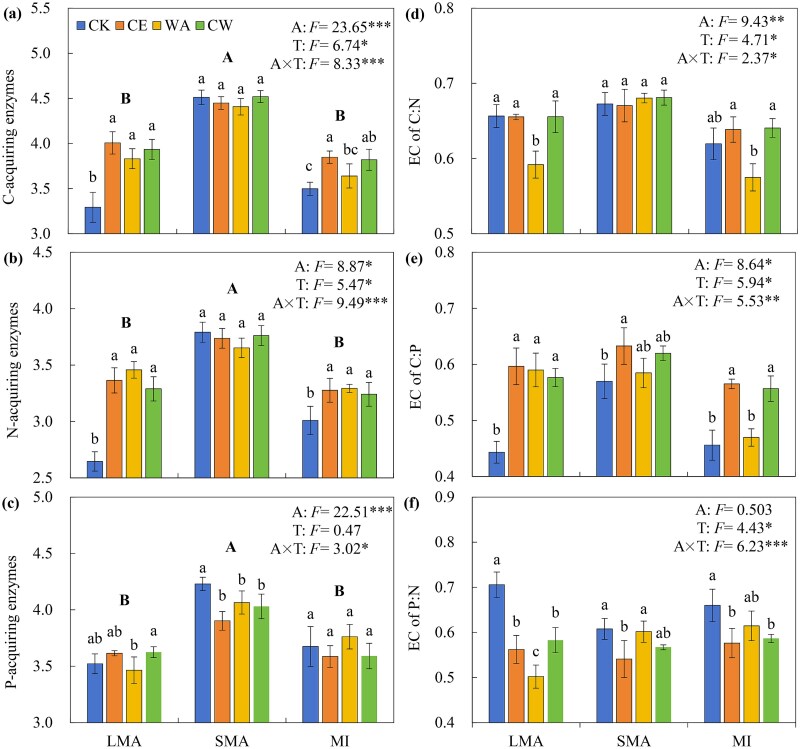
Enzyme activities and enzymatic stoichiometry (EC) in soil aggregates under climate change. Potential enzyme activities were normalized as those of C-acquiring enzymes (a), N-acquiring enzymes (b), P-acquiring enzymes (c), C: N enzyme ratio (d), C: P enzyme ratio (e), and N:P enzyme ratio (f). CK, ambient control; CE, CO_2_ enrichment; WA, canopy warming; CW, CO_2_ enrichment plus canopy warming. LMA, large macro-aggregates (>2 mm); SMA, small macro-aggregates (0.25–2 mm); MI, micro-aggregates (<0.25 mm). Letters indicate statistical differences among treatments (lowercase letters) and soil aggregate fractions (uppercase letters). The values are the results of two-way ANOVA analyses on the effects of aggregate fractions (A), climate change treatments (T), and their interactions (A × T). The asterisks “^***^,” “^**^,” and “^*^” indicate significant levels with at *P* < .001, *P* < .01, and *P* < .05, respectively.

Climate change treatment and aggregate size affected soil enzyme activities (*P* < .05, Fig. 5 and Fig. S6). Elevated CO_2_ and warming significantly increased the activities of microbial enzymes involved in C- (BG, CB, and XYL) and N-acquisition (NAG and LAP) in large macroaggregates and microaggregates but significantly decreased P-acquiring enzyme activity in small macroaggregates. Aggregate sizes had significant impact on the distribution of C-, N-, and P-acquiring enzymes with the highest values in small macroaggregates (Fig. 5a–c).

Climate change treatments and aggregates showed a significant interactive effect on enzyme stoichiometry (*P* < .05, Fig. 5d and e). Elevated CO_2_ significantly increased the C:P enzyme ratio, more pronounced in large macroaggregates. Conversely, the P:N enzyme ratio decreased in each aggregate fraction. Warming significantly increased the C:P enzyme ratio but decreased the P:N enzyme ratio only in large macroaggregates. Across all treatments, small macroaggregates exhibited higher total C:N and C:P enzyme ratios and lower P:N enzyme ratios compared to other aggregate fractions.

### Correlations among microbial parameters, enzyme activities, and soil nutrients

**Figure 6:**
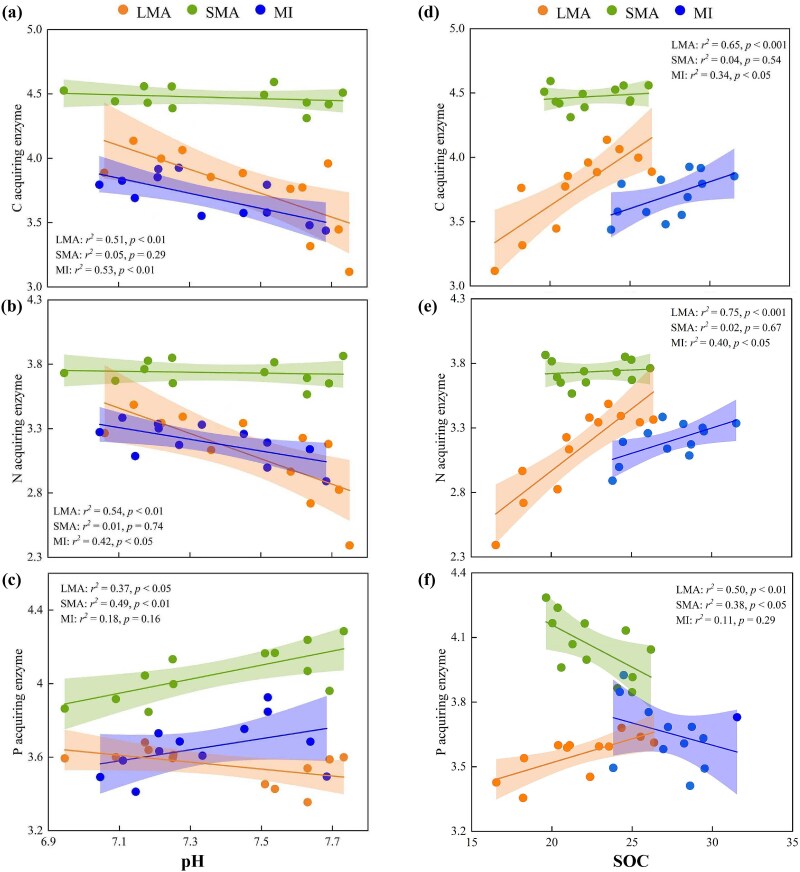
Relationships between the potential enzyme activities with soil pH (a–c) and SOC (d–f) in soil aggregates. Potential enzyme activities include those of C-acquiring enzymes, N-acquiring enzymes, and P-acquiring enzymes. The shaded bands show a 95% pointwise confidence interval of the linear regression model. SOC, soil organic carbon; LMA, large macro-aggregates (>2 mm); SMA, small macro-aggregates (0.25–2 mm); MI, micro-aggregates (<0.25 mm).

**Figure 7:**
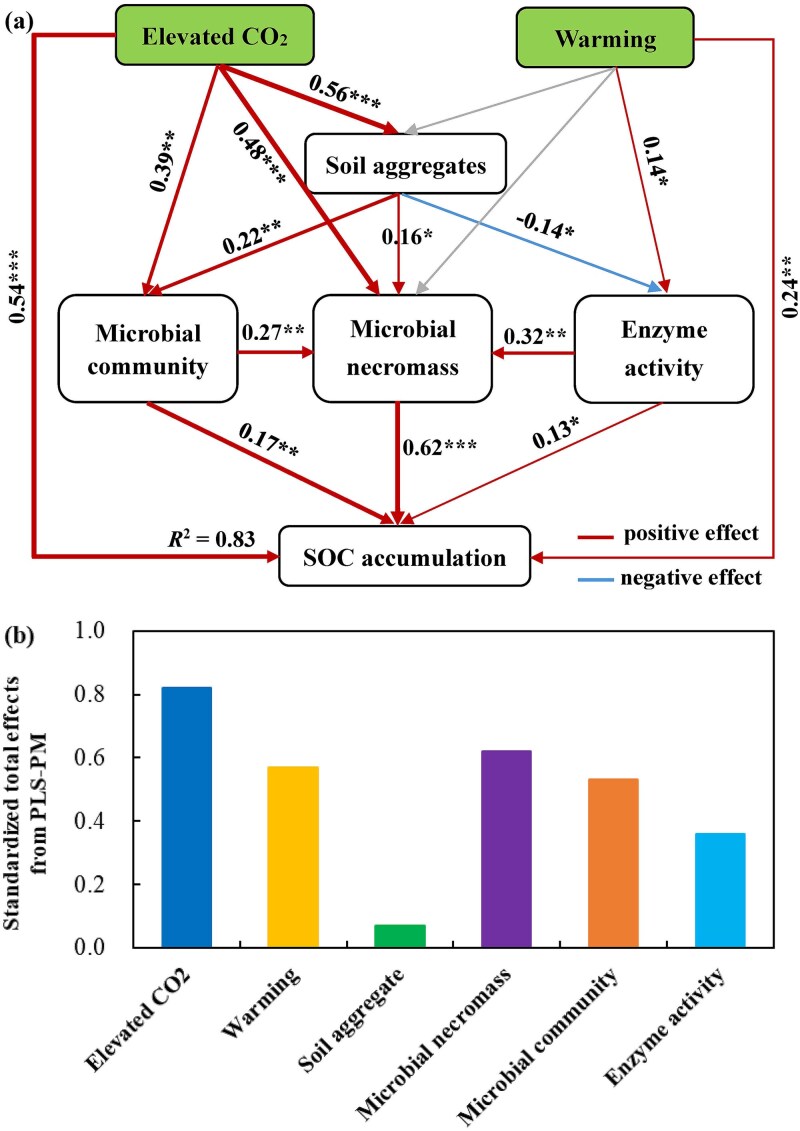
The partial least squares path models (PLS-PM) illustrating the direct and indirect effects of elevated CO_2_ and warming on SOC accumulation across soil aggregates (a) and standardized total effects derived from PLS-PM depicted above (b). Number in the arrows represent standardized path coefficients. *R*^2^ denotes the proportion of variance in the dependent variable in the model. Asterisks indicate significant relationships. Soil aggregate was characterized by the proportion of microaggregates and small and large aggregates. Microbial necromass was defined by bacterial necromass and fungal necromass. Microbial community was assessed through alpha-diversity indices and network properties. Enzyme activity was characterized by β-glucosidase, β-cellobiosidase, β-xylosidase, *N*-acetyl-β-glucosaminidase, leucine aminopeptidase, and phosphatase.

Both SOC and TP are key environmental factors regulating the microbial community in soil aggregate fractions (Fig. S7). Microbial diversities and gene abundances in large macroaggregates and microaggregates had positive relationships with SOC and TN. Still, they were negatively related to soil pH and TP. The opposite trend was observed for the small macroaggregates (Fig. S7). In large macroaggregates, the enzymes involved in C-cycling were positively correlated with fungal and bacterial diversity and abundance in the microaggregates. The activities of enzymes involved in C and N acquisition in large macroaggregates and microaggregates were positively correlated with SOC but negatively with soil pH (Fig. 6 and Fig. S8). The fungal and microbial necromass C in large and small aggregates were positively correlated with SOC and negatively correlated with soil pH (Fig. S9). Further analysis indicated that climate change was the most important variable influencing SOC, followed by microbial necromass and microbial community (Fig. 7).

## Discussion

### Soil nutrients and enzyme activities within aggregates under elevated CO_2_ and warming

The SOC and TN concentrations increased with decreasing soil aggregate size, mainly because microaggregates occupied the most significant proportion of aggregates (35.4%–50.2%, Table 1). The nutrient dynamics in the soil aggregate fractions can reflect the contribution of each aggregate size to the nutrient accumulation in bulk soil [17, 38]. Our study further suggests that long-term elevated CO_2_ and warming significantly increase SOC and TN contents across all three aggregate fractions (Tables S1–S3). This corroborates the findings from our previous studies at the same paddy field, which found increased SOC and TN accumulation in bulk and rhizosphere soils [18, 19, 39]. The increase in SOC under elevated CO_2_ may be associated with enhanced plant productivity and root exudation inputs [13, 40]. The elevated CO_2_ also had a significant impact on soil aggregate distribution, with the increased proportion of large macroaggregates and a decreased in microaggregates (Table 1). This change in distribution is partly due to the higher activities of C- and N-acquiring enzymes under elevated CO_2_ in large macroaggregates and microaggregates (Fig. 5), as these enzyme activities play an important role in the transformation and preservation of aggregates [30]. In addition, soil aggregate formation is affected by the secretion of extracellular polymeric sources [6, 8]. In this study, elevated CO_2_ significantly increased microbial abundance, especially fungal abundance in large macroaggregates. Similarly, greater labile and easily decomposable C inputs under elevated CO_2_ increased soil aggregation and SOC sequestration within aggregates [20, 41]. However, warming can increase or decrease soil aggregation and aggregate-associated C, and soil moisture can limit the differences and affect enzyme activities [18, 42]. In general, elevated CO_2_ and warming increased potential enzyme activities, accelerated microbial growth and soil mineralization, and thus increased the decomposition of labile carbon substrates by priming effects [23, 26]. Even though elevated CO_2_ and warming significantly increased C- and N-acquiring enzyme activities in our study, it had positive effect on SOC concentration. The increased C-acquiring enzyme activities (e.g. BG, CB, and XYL) may stimulate the decomposition of labile carbon sources. As a result, this portion of decomposed organic carbon was compensated by increasing plant residue and microbial necromass inputs under elevated CO_2_ and warming, leading to SOC accumulation. Additionally, the aggregate structure can provide the protection for organic carbon, limiting microbial access, and thereby enhancing SOC stability and accumulation.

In response to altered biotic and abiotic conditions, soil nutrients in soil aggregates differed in their responses to elevated CO_2_ and warming, indicating that combined elevated CO_2_ and warming can increase soil C and N accumulation but reduce soil pH and TP levels (Tables S1–S3). Small changes in soil pH can affect nutrient dynamics by altering plant nutrient inputs and microbial nutrient utilization [43, 44]. We have found that Soil pH played an important role in microbial necromass accumulation and was negatively correlated with the activities of C- and N-acquiring enzymes in large macroaggregates and microaggregates (Fig. 6 and Fig. S8) [26]. As a major environmental factor, soil pH also regulates microbial communities and activities and, consequently, potentially alters microbial necromass accumulation [45, 46]. Previous studies reported that high soil moisture and low pH in flooded paddy field can increase the accumulation of soil organic matter and nutrients under elevated CO_2_ and warming conditions [26, 43]. An increased availability of soil N can result in a microbial C use efficiency, stimulating the enzymes involved in C decomposition [47, 48]. On the same site, elevated CO_2_ and warming increased total N mineralization and mineral N production [49]. Therefore, the environmental conditions within soil aggregates under elevated CO_2_ and warming enhanced soil C and N accumulation. In this study, we have observed that fungal diversity and abundance contribute more to SOC and TN accumulation in large macroaggregates than bacterial community. The higher fungal diversity and abundance increased aggregate formation via mycelium, which has a greater capacity to decompose complex macromolecules, resulting the physical protection of nutrients and enhance their sequestration [5, 50]. The levels of microbial functional genes involved in C- and N-degradation were increased under elevated CO_2_ and warming [51, 52], which had a direct positive impact on soil C and N sequestration within soil aggregates. Thus, increased enzyme activities under climate change could stimulate the microbially mediated decomposition of organic matter, which further alters the distribution of nutrients within soil aggregates. However, elevated CO_2_ and warming decreased the soil TP level within soil aggregates (Tables S1–S3). Plant P uptake is higher under elevated CO_2_ and warming due to higher root biomass and exudates [53, 54], which increase soil P mineralization and availability. The effects of climate change on microbial communities and enzyme activities vary among soil aggregate sizes, resulting in altered soil P cycling and fraction distribution [18, 55]. This makes it important to quantify soil aggregation and nutrient levels within soil aggregates when evaluating microbial communities and their feedback to climate change, especially under elevated CO_2_ and warming.

### Response of microbial necromass within aggregates to elevated CO_2_ and warming

In this study, our results showed that elevated CO_2_ and warming explained the majority of the variation in bacterial and fungal communities in aggregates (Figs 2 and 7). Nevertheless, the aggregate effect on microbial compositions remained significant [56]. Soil aggregates provide unique habitats for microbial groups, which closely related to phylogeny in the same aggregate size and exhibited distinct climatic adaptations among the three aggregate sizes [57, 58]. The alpha diversities of microbial communities in large macroaggregates and microaggregates were significantly increased by elevated CO_2_, while were reduced in small macroaggregates (Table S6). These results support our hypothesis that microbial communities display different responses across the three aggregate fractions. In addition, the fungal diversities in large macroaggregates were positively correlated with SOC and negatively related with soil pH. The reduced soil pH caused by elevated CO_2_ may contribute to the environmental filtering, and the major fungal species are favored in acidic environment [22]. Previous studies also found that elevated CO_2_ increase SOC and microbial biomass by enhancing plant production and root exudations, leading to increase microbial growth [59, 60]. Soil nutrients are the key factors for shifting microbial structure in different aggregate fractions [24, 56]. Here, we identified soil nutrient variables for shaping microbial structure in aggregates, which were SOC and TP for bacteria, and SOC, TP, and TN for fungi (Fig. S7). Microbial community assembly processes may alter microbial complexity by imposing constraints on microbial taxa [9]. In this study, elevated CO_2_ reduced bacterial network complexity but increased fungal network complexity, suggesting different ecological strategies. Bacteria adopted stress-tolerant, low-interaction communities, while fungi capitalized on enhanced carbon inputs [11, 61]. Along with increased soil nutrients and reduced soil pH, elevated CO_2_ and warming increased the abundance of fungal pathogens and saprotrophs across aggregates (Fig. S3b). The enhanced number of pathogen and saprotroph may result from the activation of the energy metabolism and amino acid and glycan biosynthesis [18, 26]. Pathogen proliferation could disrupt beneficial microbial interactions through toxin production [46, 62], while saprotroph dominance may enhance mineralization, altering nutrient cycling [4, 63]. In contrast, the abundance of arbuscular mycorrhizal fungi decreased under long-term elevated CO_2_ and warming (Fig. S3b). The abundance of mycorrhizal fungi should theoretically decline with reducing plant diversity. Therefore, the biotic stresses of increased pathogens and decreased plant diversity under climate change inhibit the colonization of fungal symbionts [63].

In this study, we found that elevated CO_2_ significantly increased microbial necromass C, especially in large and small macroaggregates (Fig. 2). The accumulation of microbial necromass depends on the balance of microbial degradation and production. Soil microbial necromass increases quickly in response to plant residue inputs, which were converted into microbial polymers and finally form microbial necromass [17]. It had been reported that elevated CO_2_ can increase rice biomass and root exudation [21, 24], which increase microbial necromass accumulation by promoting microbial growth and anabolism. Our results showed that microbial abundance and enzyme activities of C- and N-acquiring increased under elevated CO_2_ (Tables S1–S3 and Fig. 2). The increased enzyme activities decompose the depolymerization of large organic compounds into monomers and oligomers, which can provide substrate for microbial growth and reproduction. We further found that elevated CO_2_ and warming significantly increased fungal and bacterial necromass C and its contribution to SOC in macroaggregates and microaggregates, respectively (Fig. 2 and Fig. S1). The different responses of microbial necromass accumulation to elevated CO_2_ and warming among aggregate sizes may due to the following reasons: Macroaggregates favored fungal communities, possibly due to their hyphal networks and ability to decompose complex organic matter, whereas bacteria dominated microaggregates, reflecting their preference for labile substrates [4, 62]. Fungal species typically have higher biomass C:N ratio and greater C utilization efficiency, contributing mainly to microbial colonization of plant litter compared to bacteria. The increased plant productivity and root exudate inputs under elevated CO_2_ could increase the fungal contribution to microbial communities [22]. In addition, soil pH has adversely effect on amino sugars and lower soil pH is conducive to enhancing fungal growth and necromass [64], which was supported by negative correlation between fungal necromass C and pH in macroaggregates (Fig. S9a). We previously found that warming increased the rice root exudates including sugars and amino acids [21], which accelerated bacterial growth, proliferation, and turnover, thereby enhancing bacterial necromass accumulation [65]. Moreover, we found that the change in bacterial network was opposite to that of fungi (Fig. 4), with their different growth strategies and ecological niches which may lead to different responses of bacterial and fungal necromass to climate changes [24, 66].

## Conclusions

Our results from a decade-long field experiment provide evidence that soil aggregate size mediates soil nutrients and microbial responses to climate change. Elevated CO_2_ and warming significantly increased fungal and bacterial abundances, as well as the potential activities of C- and N-potential enzyme in large macroaggregate and microaggregates. Conversely, the P-acquiring enzyme activities and the stoichiometry for P:N enzyme ratio was significantly decreased by elevated CO_2_ and warming. However, microbial necromass responses varied among aggregate sizes. Fungal necromass in macroaggregates showed higher sensitivity to elevated CO_2_, while bacterial necromass within microaggregates is more susceptible to warming. The SOC content is positively correlated with microbial necromass C (mainly fungal necromass), which was enhanced by low pH in all three aggregates. These results suggest that elevated CO_2_ and warming increase microbial activities, accelerating the decomposition of SOC. However, the microbial necromass can enhance the stability of SOC, leading to the accumulation of SOC. Although significant increases in soil C and N contents, elevated CO_2,_ and warming limited soil P concentration, resulting in a risk of nutrient imbalance. Given the important role of soil aggregate distribution in regulating microbial responses to climate change, further research is required to incorporate these processes into ecosystem models for accurately predict soil C cycling under future climate scenarios.

## Supplementary Material

Supplementary_material_ycag206

## Data Availability

Data will be made available on request.
