# Supplementary material for: Soil aggregate and microbial traits mediate soil organic carbon accumulation in a paddy field under long-term elevated CO2 and warming
Source: ISME Commun. 2026 Jul 14;6(1):ycag206. doi: 10.1093/ismeco/ycag206 (PMC13477214; doi:10.1093/ismeco/ycag206)
Supplement: Supplementary file 1 [file supplementary_material_ycag206.docx]

**Supporting Information**

**Title****: Soil aggregate and microbial traits mediate soil organic carbon accumulation in a paddy field under long-term elevated CO_2_ and warming**

Yuan Liu^1*^, Mengting Li^1^, Jiayi Xiong^1^, Feng Li^1^, Cheng Liu^2*^, Xiaoyu Liu^3^

^1^Anhui Province Key Laboratory of Pollutant Sensitive Materials and Environmental Remediation, College of Life Science, Huaibei Normal University, Huaibei 235000, Anhui, China

^2^Institute of Eco-environmental Research, Zhejiang University of Science and Technology, Hangzhou 310023, China

^3^Institute of Resources, Ecosystem and Environment of Agriculture, Nanjing Agricultural University, 1 Weigang, Nanjing 210095, China

**Running title:** **Soil aggregate mediated microbial responses to climate change**

*Corresponding author. Yuan Liu, Anhui Province Key Laboratory of Pollutant Sensitive Materials and Environmental Remediation, College of Life Science, Huaibei Normal University, No. 100 Dongshan Road, Huaibei 235000, Anhui, China. Email: [liuyuan0813@aliyun.com](mailto:liuyuan0813@aliyun.com); Cheng Liu, Institute of Eco-environmental Research, Zhejiang University of Science and Technology, No. 318 Liuhe Road, Hangzhou 310023, China. Email: [chengliu01@zust.edu.cn](mailto:chengliu01@zust.edu.cn;)

**Supplementary Materials and methods**

**Soil amino sugars analysis**

Soil amino sugars were analyzed following the classic method according to (Liang et al., 2012). Briefly, about 0.5 g air-dried soil was hydrolyzed with 10 mL of 6 M HCl at 105 ℃ for 8 h. Then, 100 µg of myo-inositol was injected into the cooled samples as a surrogate standard, which was filtered and dried via rotary evaporation at 50-53 ℃ under reduced pressure. The residues were re-dissolved in 5 mL of Milli-Q water and adjusted to pH 6.6-6.8 with 1 M potassium hydroxide. After discarding the precipitate by centrifugation, the supernatant was dried completely by rotary evaporation. The resultant residues were re-dissolved in absolute methanol and transferred to a vial to dry them under a gentle stream of N_2_ at 45 ℃. Amino sugars were redissolved in 1 mL Milli-Q water, with an additional 100 µg of recovery standard (*N*-methylglucamine), and subsequently freeze-dried overnight. The purified amino sugars were treated with 0.3 mL derivatization reagents (32 mg mL^-1^ hydroxylamine hydrochloride and 40 mg mL^-1^ 4-dimethylamino-pyridine in pyridine-methanol at a 4:1 vol ratio) at 75-80 ℃ for 30 min to obtain the aldononitrile derivatives. After cooling to room temperature, the derivatives were further acetylated with 1 mL of acetic anhydride at 75-80 ◦C for 20 min and mixed with 1.5 mL of dichloromethane. Excess derivatization reagents were thoroughly removed by extraction with HCl (1 M) and Milli-Q water. The amino sugar derivatives concentrated by N_2_ were dissolved in 500 µL of a mixture of hexane and ethyl acetate solvent (*v*:*v* = 1:1) for analysis.

Total amino sugar contents were calculated as the sum of muramic acid (MurA), glucosamine (GlcN) and galactosamine (GalN). Bacterial necromass C and fungal necromass C were calculated by the following formula (Hu et al., 2024):

Bacterial necromass C (BNC) = MurA × 31.3,

Fungal necromass C (FNC) = (GlcN/179.2 – 2 × MurA/251.2) × 179.2 × 10.8,

Microbial necromass C (MNC) = BNC + FNC

Where 31.3 is the conversion factor of bacterial MurA to bacterial necromass C; 10.8 is the conversion factor of fungal GlcN to fungal necromass C; 179.2 and 251.2 are the molecular weight of GlcN and MurA, respectively. Total microbial necromass C was summarized as bacterial necromass C and fungal necromass C.

**References**

Liang, C., Read, H.W., Balser, T.C., 2012. GC-Based detection of aldononitrile acetate derivatized glucosamine and muramic acid for microbial residue determination in soil. Jove-Journal of Visualized Experiments 63, e3767.

Zhang, X., Amelung, W., 1996. Gas chromatographic determination of muramic acid, glucosamine, mannosamine, and galactosamine in soils. Soil Biology and Biochemistry 28, 1201–1206.

| Aggregates | Treatment | pH  (H_2_O) | | SOC  (g kg^−1^) | | TN  (g kg^−1^) | | TP  (g kg^−1^) | | Bacterial abundance  (10^9^ copies g^−1^ soil) | | Fungal abundance  (10^7^ copies g^−1^ soil) | |
| --- | --- | --- | --- | --- | --- | --- | --- | --- | --- | --- | --- | --- | --- |
| LMA  (> 2 mm) | CK | 7.67 ± 0.08 aA | | 18.76 ± 1.02 bB | | 1.43 ± 0.07 bA | | 2.28 ± 0.15 aA | | 2.23 ± 0.54 cA | | 10.46 ± 2.14 cA | |
|  | CE | 7.23 ± 0.05 bA | | 24.14 ± 1.36 aB | | 1.69 ± 0.08 aA | | 2.07 ± 0.09 abA | | 2.84 ± 0.32 bA | | 23.18 ± 2.05 aA | |
|  | WA | 7.56 ± 0.05 aA | | 21.57 ± 1.18 abB | | 1.49 ± 0.06 bA | | 1.94 ± 0.11 bA | | 2.67 ± 0.42 bA | | 12.81 ± 1.75 cA | |
|  | CW | 7.17 ± 0.06 bA | | 24.76 ± 1.32 aB | | 1.73 ± 0.05 aB | | 1.86 ± 0.08 bA | | 3.21 ± 0.45 aA | | 19.19 ± 2.18 bA | |
| SMA  (0.25-2 mm) | CK | 7.62 ± 0.08 aA | | 19.34 ± 0.87 bB | | 1.48 ± 0.10 aA | | 1.85 ± 0.11 aB | | 1.86 ± 0.23 bB | | 1.32 ± 0.45 bB | |
|  | CE | 7.16 ± 0.11 bA | | 23.13 ± 1.16 aB | | 1.72 ± 0.09 aA | | 1.46 ± 0.09 bB | | 2.57 ± 0.43 aA | | 1.86 ± 0.31 aB | |
|  | WA | 7.65 ± 0.07 aA | | 21.69 ± 1.03 bB | | 1.54 ± 0.12 aA | | 1.37 ± 0.09 bB | | 2.53 ± 0.31 aA | | 1.47 ± 0.43 bB | |
|  | CW | 7.08 ± 0.10 bA | | 25.14 ± 0.81 aB | | 1.76 ± 0.11 aB | | 1.35 ± 0.12 bB | | 2.71 ± 0.43 aB | | 1.75 ± 0.33 aB | |
| MI  (< 0.25 mm) | CK | 7.69 ± 0.11 aA | | 25.67 ± 1.34 bA | | 1.60 ± 0.08 bA | | 1.41 ± 0.10 aC | | 2.06 ± 0.37 bAB | | 1.16 ± 0.44 cB | |
|  | CE | 7.08 ± 0.09 bA | | 29.15 ± 1.25 aA | | 1.88 ± 0.12 aA | | 1.02 ± 0.09 bC | | 2.88 ± 0.32 aA | | 1.95 ± 0.34 aB | |
|  | WA | 7.59 ± 0.12 aA | | 26.67 ± 1.13 bA | | 1.62 ± 0.08 bA | | 0.95 ± 0.12 bC | | 2.81 ± 0.42 aA | | 1.56 ± 0.28 bcB | |
|  | CW | 7.12 ± 0.08 bA | | 29.76 ± 1.01 aA | | 1.98 ± 0.13 aA | | 0.93 ± 0.09 bC | | 2.96 ± 0.34 aAB | | 1.67 ± 0.32 abB | |
| *Factor (Df)* | | *F* | *p* | *F* | *p* | *F* | *p* | *F* | *p* | *F* | *p* | *F* | *p* |
| *Aggregates (2)* | | *6.36* | *** | *43.56* | ***** | *5.57* | *** | *107.56* | ***** | *1.26* | *0.324* | *38.77* | ***** |
| *Treatments (3)* | | *49.88* | ***** | *19.54* | ***** | *7.44* | *** | *22.14* | **** | *4.86* | *** | *14.46* | **** |
| *Aggregates × Treatments (6)* | | *1.33* | *0.256* | *1.13* | *0.322* | *3.96* | *** | *4.32* | **** | *3.37* | *** | *6.71* | **** |

**Table S1** Soil properties and microbial abundance of soil aggregates in 2020 under long-term climate change.

CK, ambient control; CE, CO_2_ enrichment; WA, canopy warming; CW, CO_2_ enrichment plus canopy warming; LMA, large macro-aggregates (> 2 mm); SMA, small macro-aggregates (0.25–2 mm); MI, micro-aggregates (< 0.25 mm). SOC, soil organic carbon; TN, total nitrogen; TP, total phosphorus. Letters indicate statistical differences (*p* < 0.05) among treatments in each aggregate size (lowercase letters) and aggregate sizes in each treatment (uppercase letters). The values are two-way ANOVA analyses on effects of the aggregate fractions, climate change treatments, and their interactions. The asterisks ‘***’, ‘**’, and ‘*’ indicate significant levels with *p* < 0.001, *p* < 0.01, and *p* < 0.05, respectively.

| Aggregates | Treatment | pH  (H_2_O) | | SOC  (g kg^−1^) | | TN  (g kg^−1^) | | TP  (g kg^−1^) | | Bacterial abundance  (10^9^ copies g^−1^ soil) | | Fungal abundance  (10^7^ copies g^−1^ soil) | |
| --- | --- | --- | --- | --- | --- | --- | --- | --- | --- | --- | --- | --- | --- |
| LMA  (> 2 mm) | CK | 7.75 ± 0.05 aA | | 17.36 ± 1.37 bB | | 1.32 ± 0.07 cA | | 2.06 ± 0.07 aA | | 4.78 ± 0.28 bA | | 12.54 ± 2.03 cA | |
|  | CE | 7.15 ± 0.06 bA | | 24.46 ± 1.14 aB | | 1.58 ± 0.07 abB | | 1.74 ± 0.04 bA | | 5.56± 0.58 aA | | 25.85 ± 2.58 aA | |
|  | WA | 7.70 ± 0.07 aA | | 19.86 ± 1.36 bB | | 1.51 ± 0.05 bA | | 1.86 ± 0.06 abA | | 4.41 ± 0.30 bA | | 18.84 ± 1.45 bA | |
|  | CW | 7.16 ± 0.07 bA | | 24.16 ± 1.27 aB | | 1.75 ± 0.08 aA | | 1.67 ± 0.05 bA | | 5.37 ± 0.37 aA | | 23.29 ± 2.15 aA | |
| SMA  (0.25-2 mm) | CK | 7.67 ± 0.07 aA | | 19.15 ± 1.04 bB | | 1.48 ± 0.08 bA | | 1.83 ± 0.11 aB | | 2.66 ± 0.32 bB | | 1.61 ± 0.22 bB | |
|  | CE | 7.07 ± 0.06 cA | | 24.76 ± 1.25 aB | | 1.77 ± 0.09 aAB | | 1.53 ± 0.08 bB | | 3.54 ± 0.38 aB | | 2.13 ± 0.32 aB | |
|  | WA | 7.45 ± 0.09 bA | | 22.74 ± 0.85 abB | | 1.68 ± 0.12 abA | | 1.45 ± 0.07 bcB | | 2.98 ± 0.35 bB | | 1.98 ± 0.43 aB | |
|  | CW | 7.14 ± 0.06 cA | | 25.85 ± 0.94 aB | | 1.85 ± 0.11 aA | | 1.28 ± 0.12 cB | | 3.76 ± 0.36 aAB | | 2.04 ± 0.25 aB | |
| MI  (< 0.25 mm) | CK | 7.65 ± 0.09 aA | | 24.86 ± 1.86 cA | | 1.51 ± 0.10 bA | | 1.54 ± 0.09 aC | | 2.35 ± 0.43 bB | | 1.32 ± 0.32 bB | |
|  | CE | 7.06 ± 0.11 bA | | 28.94 ± 1.32 abA | | 1.86 ± 0.11 aA | | 1.13 ± 0.07 bC | | 3.05 ± 0.31 aB | | 1.97 ± 0.18 aB | |
|  | WA | 7.51 ± 0.13 aA | | 26.25 ± 0.89 bcA | | 1.64 ± 0.09 bA | | 1.08 ± 0.11 bcC | | 2.87 ± 0.47 aB | | 1.58 ± 0.31 abB | |
|  | CW | 7.02 ± 0.08 bB | | 29.86 ± 1.51 aA | | 1.91 ± 0.12 aA | | 0.97 ± 0.06 cC | | 3.23 ± 0.38 aB | | 1.86 ± 0.24 aB | |
| *Factor (Df)* | | *F* | *p* | *F* | *p* | *F* | *p* | *F* | *p* | *F* | *p* | *F* | *p* |
| *Aggregates (2)* | | *6.85* | *** | *42.85* | ***** | *5.77* | *** | *102.02* | ***** | *1.72* | *0.262* | *36.49* | ***** |
| *Treatments (3)* | | *52.46* | ***** | *16.32* | ***** | *7.61* | *** | *19.12* | **** | *4.56* | *** | *12.07* | **** |
| *Aggregates × Treatments (6)* | | *1.28* | *0.269* | *1.51* | *0.288* | *3.78* | *** | *4.02* | **** | *3.46* | *** | *6.47* | **** |

**Table S2** Soil properties and microbial abundance within soil aggregates in 2021.

CK, ambient control; CE, CO_2_ enrichment; WA, canopy warming; CW, CO_2_ enrichment plus canopy warming; LMA, large macro-aggregates (> 2 mm); SMA, small macro-aggregates (0.25–2 mm); MI, micro-aggregates (< 0.25 mm). SOC, soil organic carbon; TN, total nitrogen; TP, total phosphorus. Letters indicate statistical differences (*p* < 0.05) among treatments in each aggregate size (lowercase letters) and aggregate sizes in each treatment (uppercase letters). The values are two-way ANOVA analyses on effects of the aggregate fractions (A), climate change treatments (T), and their interactions (A × T). The asterisks ‘***’, ‘**’, and ‘*’ indicate significant levels with *p* < 0.001, *p* < 0.01, and *p* < 0.05, respectively.

| Aggregates | Treatment | pH  (H_2_O) | | SOC  (g kg^−1^) | | TN  (g kg^−1^) | | TP  (g kg^−1^) | | Bacterial abundance  (10^9^ copies g^−1^ soil) | | Fungal abundance  (10^7^ copies g^−1^ soil) | |
| --- | --- | --- | --- | --- | --- | --- | --- | --- | --- | --- | --- | --- | --- |
| LMA  (> 2 mm) | CK | 7.70 ± 0.06 aA | | 18.40 ± 1.63 cB | | 1.39 ± 0.05 bA | | 2.17 ± 0.12 aA | | 2.10 ± 0.34 bA | | 9.46 ± 1.33 cA | |
|  | CE | 7.14 ± 0.08 cA | | 25.15 ± 1.44 aB | | 1.62 ± 0.06 aB | | 1.92 ± 0.06 bA | | 2.77 ± 0.18 aA | | 19.8 ± 2.76 aA | |
|  | WA | 7.63 ± 0.05 aA | | 20.51 ± 1.13 bcB | | 1.45 ± 0.09 bA | | 1.86 ± 0.09 bA | | 2.13 ± 0.30 bB | | 11.8 ± 1.69 bcA | |
|  | CW | 7.36 ± 0.09 bA | | 22.80 ± 1.62 abB | | 1.69 ± 0.09 aB | | 1.83 ± 0.06 bA | | 2.52 ± 0.25 abAB | | 17.9 ± 1.91 abA | |
| SMA  (0.25-2 mm) | CK | 7.63 ± 0.10 aA | | 20.01 ± 0.36 bB | | 1.54 ± 0.07 aA | | 1.64 ± 0.07 aB | | 1.70 ± 0.11 bB | | 1.11 ± 0.14 bB | |
|  | CE | 7.13 ± 0.16 bA | | 23.75 ± 1.45 aB | | 1.66 ± 0.11 aB | | 1.37 ± 0.10 bB | | 2.64 ± 0.29 aA | | 1.53 ± 0.11 aB | |
|  | WA | 7.61 ± 0.09 aA | | 21.31 ± 0.73 bB | | 1.48 ± 0.10 aA | | 1.06 ± 0.06 cB | | 2.42 ± 0.35 aAB | | 1.38 ± 0.13 abB | |
|  | CW | 7.17 ± 0.08 bB | | 25.27 ± 0.81 aB | | 1.65 ± 0.13 aB | | 1.11 ± 0.05 cB | | 2.13 ± 0.38 abB | | 1.44 ± 0.13 aB | |
| MI  (< 0.25 mm) | CK | 7.61 ± 0.09 aA | | 25.11 ± 0.89 bA | | 1.54 ± 0.09 bA | | 1.46 ± 0.08 aC | | 1.60 ± 0.46 bB | | 1.15 ± 0.27 bB | |
|  | CE | 7.14 ± 0.12 cA | | 28.48 ± 1.12 aA | | 1.98 ± 0.10 aA | | 0.95 ± 0.06 bC | | 2.94 ± 0.22 aA | | 2.01 ± 0.21 aB | |
|  | WA | 7.43 ± 0.09 bA | | 27.75 ± 1.19 abA | | 1.47 ± 0.08 bA | | 0.89 ± 0.05 bB | | 2.82 ± 0.31 aA | | 1.61 ± 0.18 abB | |
|  | CW | 7.19 ± 0.04 cB | | 29.75 ± 1.36 aA | | 1.90 ± 0.05 aA | | 0.90 ± 0.05 bC | | 3.12 ± 0.44 aA | | 1.79 ± 0.19 aB | |
| *Factor (Df)* | | *F* | *p* | *F* | *p* | *F* | *p* | *F* | *p* | *F* | *p* | *F* | *p* |
| *Aggregates (2)* | | *6.38* | *** | *38.85* | ***** | *4.33* | *0.058* | *113.22* | ***** | *1.59* | *0.279* | *31.35* | ***** |
| *Treatments (3)* | | *45.84* | ***** | *17.31* | ***** | *7.92* | *** | *20.35* | **** | *5.28* | *** | *10.34* | **** |
| *Aggregates × Treatments (6)* | | *1.44* | *0.238* | *1.26* | *0.309* | *3.64* | *** | *4.14* | **** | *3.12* | *** | *5.91* | **** |

**Table S3** Soil properties and microbial abundance within soil aggregates in 2023.

CK, ambient control; CE, CO_2_ enrichment; WA, canopy warming; CW, CO_2_ enrichment plus canopy warming; LMA, large macro-aggregates (> 2 mm); SMA, small macro-aggregates (0.25–2 mm); MI, micro-aggregates (< 0.25 mm). SOC, soil organic carbon; TN, total nitrogen; TP, total phosphorus. Letters indicate statistical differences (*p* < 0.05) among treatments in each aggregate size (lowercase letters) and aggregate sizes in each treatment (uppercase letters). The values are two-way ANOVA analyses on effects of the aggregate fractions (A), climate change treatments (T), and their interactions (A × T). The asterisks ‘***’, ‘**’, and ‘*’ indicate significant levels with *p* < 0.001, *p* < 0.01, and *p* < 0.05, respectively.

|  | pH | | SOC | | TN | | TP | | Bacterial abundance | | Fungal abundance | |
| --- | --- | --- | --- | --- | --- | --- | --- | --- | --- | --- | --- | --- |
| Factor *(Df)* | *F* | *p* | *F* | *p* | *F* | *p* | *F* | *p* | *F* | *p* | *F* | *p* |
| Aggregates (2) | *0.124* | *0.885* | *6.132* | *** | *1.316* | *0.315* | *14.610* | **** | *3.786* | *0.064* | *31.843* | ***** |
| Treatments (3) | *4.453* | *** | *9.804* | **** | *7.004* | **** | *0.774* | *0.540* | *2.471* | *0.136* | *0.138* | *0.934* |
| Year (2) | *0.734* | *0.494* | *0.165* | *0.849* | *2.184* | *0.141* | *3.181* | *0.069* | *119.417* | ***** | *31.172* | ***** |
| Aggregates × Treatments (6) | *1.176* | *0.327* | *2.186* | *0.105* | *1.965* | *0.134* | *6.643.* | *** | *2.854* | *0.064* | *11.964* | **** |
| Aggregates × Year (4) | *0.566* | *0.691* | *1.943* | *0.147* | *1.553* | *0.230* | *2.343* | *0.076* | *36.006* | ***** | *22.444* | ***** |
| Treatments × Year (6) | *1.550* | *0.225* | *0.945* | *0.491* | *2.266* | *0.090* | *0.361* | *0.793* | *0.157* | *0.985* | *0.162* | *0.983* |
| Aggregates × Treatments × Year (12) | *0.505* | *0.795* | *1.096* | *0.325* | *0.876* | *0.414* | *0.413* | *0.912* | *1.452* | *0.278* | *2.045* | *0.158* |

**Table S4** Repeated measures ANOVA of soil properties and microbial abundance.

SOC, soil organic carbon; TN, total nitrogen; TP, total phosphorus. Letters indicate statistical differences (*p* < 0.05) among treatments in each aggregate size (lowercase letters) and aggregate sizes in each treatment (uppercase letters). The asterisks ‘***’, ‘**’, and ‘*’ indicate significant levels with *p* < 0.001, *p* < 0.01, and *p* < 0.05, respectively.

**Table S5** The number and Good’s coverage of sequencing of bacterial and fungal communities in soil aggregate sizes under long-term climate change.

| Aggregates |  |  | Fungi | | | Bacteria | | | |
| --- | --- | --- | --- | --- | --- | --- | --- | --- | --- |
|  |  | Number | | Good’s coverage |  | | Number | Good’s coverage |  |
| LMA  (> 2 mm) | CK | 65376 ± 1342 | | 99.81% |  | | 67238 ± 1164 | 99.95% |  |
|  | CE | 64557 ± 1664 | | 99.97% |  | | 68586 ± 1832 | 99.97% |  |
|  | WA | 66214 ± 1542 | | 99.91% |  | | 65899 ± 1658 | 99.96% |  |
|  | CW | 64874 ± 1422 | | 99.98% |  | | 67904 ± 2464 | 99.98% |  |
| SMA  (0.25-2 mm) | CK | 63122 ± 1764 | | 99.95% |  | | 66546 ± 1343 | 99.97% |  |
|  | CE | 64188 ± 1249 | | 99.89% |  | | 62512 ± 1176 | 99.97% |  |
|  | WA | 62152 ± 1535 | | 99.89% |  | | 65928 ± 1567 | 99.97% |  |
|  | CW | 63782 ± 1391 | | 99.84% |  | | 68532 ± 1271 | 99.96% |  |
| MI  (< 0.25 mm) | CK | 66014 ± 1538 | | 99.84% |  | | 67904 ± 1034 | 99.95% |  |
|  | CE | 65678 ± 1383 | | 99.98% |  | | 65712 ± 1485 | 99.96% |  |
|  | WA | 66125 ± 1650 | | 99.96% |  | | 69645 ± 1251 | 99.98% |  |
|  | CW | 63011 ± 1505 | | 99.96% |  | | 69885 ± 1708 | 99.97% |  |

CK, ambient control; CE, CO_2_ enrichment; WA, canopy warming; CW, CO_2_ enrichment plus canopy warming; LMA, large macro-aggregates (> 2 mm); SMA, small macro-aggregates (0.25–2 mm); MI, micro-aggregates (< 0.25 mm).

**Table S6** The alpha-diversity of bacterial and fungal communities in soil aggregate sizes under long-term climate change.

| Aggregates |  | Bacteria | | | | | | | | |  | | Fungi | | | | | | | | |
| --- | --- | --- | --- | --- | --- | --- | --- | --- | --- | --- | --- | --- | --- | --- | --- | --- | --- | --- | --- | --- | --- |
|  |  | Observed OTUs | | Chao1 | | ACE | | Shannon | |  | | Observed OTUs | | | Chao1 | | ACE | | Shannon | |  |
| LMA  (> 2 mm) | CK | 2454 ± 79 bB | | 2491 ± 90 aB | | 2479 ± 85 bB | | 7.16 ± 0.03 bB | |  | | 349 ± 7 bB | | | 352 ± 7 bB | | 351 ± 6 bB | | 4.58 ± 0.20 bA | |  |
|  | CE | 2683 ± 39 aA | | 2718 ± 42 aA | | 2709 ± 40 aA | | 7.27 ± 0.02 aB | |  | | 509 ± 59 aAB | | | 524 ± 70 aAB | | 521 ± 69 aAB | | 4.85 ± 0.11 aB | |  |
|  | WA | 2463 ± 91 bB | | 2503 ± 90 aB | | 2489 ± 94 bB | | 7.16 ± 0.08 bB | |  | | 453 ± 46 aA | | | 458 ± 47 aA | | 458 ± 47 aA | | 4.45 ± 0.20 bA | |  |
|  | CW | 2536 ± 5 abA | | 2568 ± 6 aA | | 2563 ± 3 abA | | 7.21 ± 0.01 abA | |  | | 441 ± 18 aB | | | 447 ± 19 aB | | 446 ± 19 abB | | 4.60 ± 0.11 bA | |  |
| SMA  (0.25-2 mm) | CK | 2803 ± 64 aA | | 2847 ± 65 aA | | 2834 ± 63 aA | | 7.35 ± 0.02 aA | |  | | 398 ± 57 aB | | | 401 ± 61 aB | | 400 ± 59 aB | | 4.92 ± 0.15 aA | |  |
|  | CE | 2637 ± 49 aA | | 2666 ± 50 aA | | 2661 ± 52 aA | | 7.25 ± 0.01 bB | |  | | 398 ± 41 aB | | | 401 ± 41 aB | | 400 ± 41 aB | | 4.86 ± 0.05 aB | |  |
|  | WA | 2795 ± 35 aA | | 2841 ± 30 aA | | 2828 ± 35 aA | | 7.29 ± 0.02 abA | |  | | 430 ± 7 aA | | | 436 ± 7 aA | | 437 ± 7 aA | | 4.34 ± 0.14 bA | |  |
|  | CW | 2691 ± 91 aA | | 2792 ± 98 aA | | 2721 ± 99 aA | | 7.26 ± 0.06 bA | |  | | 375 ± 62 aB | | | 378 ± 65 aB | | 378 ± 65 aB | | 4.90 ± 0.07 aA | |  |
| MI  (< 0.25 mm) | CK | 2527 ± 38 bB | | 2531 ± 84 bB | | 2517 ± 95 bB | | 7.22 ± 0.02 bcB | |  | | 561 ± 36 abA | | | 568 ± 39 abA | | 567 ± 38 abA | | 4.80 ± 0.08 cA | |  |
|  | CE | 2773 ± 83 aA | | 2818 ± 91 aA | | 2807 ± 89 aA | | 7.31 ± 0.02 aA | |  | | 624 ± 63 aA | | | 632 ± 65 aA | | 632 ± 68 aA | | 5.26 ± 0.02 aA | |  |
|  | WA | 2549 ± 50 bAB | | 2582 ± 48 bAB | | 2575 ± 49 bAB | | 7.18 ± 0.01 cB | |  | | 439 ± 27 bA | | | 450 ± 27 bA | | 447 ± 28 bA | | 3.93 ± 0.03 dB | |  |
|  | CW | 2678 ± 105 abA | | 2713 ± 118 abA | | 2706 ± 111 abA | | 7.25 ± 0.04 bA | |  | | 487 ± 73 abA | | | 499 ± 71 abA | | 494 ± 76 abA | | 4.93 ± 0.03 bA | |  |
| *Factor (Df)* | | *F* | *p* | *F* | *p* | *F* | *p* | *F* | *p* |  | | *F* | | *p* | *F* | *p* | *F* | *p* | *F* | *p* |  |
| *Aggregates (2)* | | *3.25* | *0.111* | *3.01* | *0.125* | *2.99* | *0.125* | *2.63* | *0.151* |  | | *4.56* | | *0.062* | *4.87* | *0.055* | *4.66* | *0.060* | *0.347* | *0.720* |  |
| *Treatments (3)* | | *0.54* | *0.669* | *0.52* | *0.684* | *0.56* | *0.660* | *0.89* | *0.496* |  | | *1.08* | | *0.427* | *1.13* | *0.409* | *1.11* | *0.414* | *5.34* | *** |  |
| *Aggregates × Treatments (6)* | | *3.66* | *** | *3.65* | *** | *3.65* | *** | *6.48* | ***** |  | | *2.10* | | *0.091* | *1.91* | *0.120* | *2.01* | *0.104* | *24.17* | ***** |  |

CK, ambient control; CE, CO_2_ enrichment; WA, canopy warming; CW, CO_2_ enrichment plus canopy warming; LMA, large macro-aggregates (> 2 mm); SMA, small macro-aggregates (0.25–2 mm); MI, micro-aggregates (< 0.25 mm). Different letters indicate statistical differences (*p* < 0.05) among different treatments in each aggregate size (lowercase letters) and different aggregate sizes in each treatment (uppercase letters). The asterisks ‘***’, ‘**’and ‘*’ indicate significant levels with at *p* < 0.001, *p* < 0.01 and *p* < 0.05, respectively.

**Table S7** The relative abundance of dominant bacterial and fungal communities at the phylum level in soil aggregate sizes under long-term climate change.

| Aggregates |  | Relative abundance (%) of the dominant bacterial phylum | | | | | | | | | | |  | Relative abundance (%) of the dominant fungal phylum | | | | | | | |
| --- | --- | --- | --- | --- | --- | --- | --- | --- | --- | --- | --- | --- | --- | --- | --- | --- | --- | --- | --- | --- | --- |
|  |  | Chloroflexi | | Proteobacteria | | Acidobacteria | | Bacteroidota | | Actinobacteria | |  | | Ascomycota | | Basidiomycota | | Mortierellomycota | | Glomeromycota | |
| LMA  (> 2 mm) | CK | 31.30 ± 1.21 cA | | 14.70 ± 0.56 aB | | 11.60 ± 0.36 bA | | 5.90 ± 0.20 aA | | 4.43 ± 0.49 cB | |  | | 36.70 ± 4.44 aB | | 23.50 ± 2.52 abA | | 4.23 ± 1.88 aB | | 10.77 ± 2.75 aA | |
|  | CE | 33.07 ± 1.27 bA | | 15.80 ± 0.56 aB | | 11.03 ± 0.21 cC | | 5.90 ± 0.56 aA | | 6.40 ± 0.85 aA | |  | | 50.27 ± 7.06 aA | | 19.10 ± 7.63 aA | | 2.47 ± 0.40 aC | | 7.10 ± 0.69 aA | |
|  | WA | 40.50 ± 0.46 aA | | 15.13 ± 0.47 aB | | 9.00 ± 0.20 dB | | 6.60 ± 0.61 aA | | 5.97 ± 0.21 abB | |  | | 55.63 ± 7.10 aA | | 15.20 ± 3.16 cA | | 5.40 ± 3.27 aA | | 6.83 ± 1.12 aA | |
|  | CW | 28.77 ± 0.31 dA | | 15.77 ± 0.81 aB | | 12.90 ± 0.17 aA | | 6.62 ± 0.40 aA | | 5.07 ± 0.32 bcA | |  | | 43.03 ± 3.54 aA | | 20.60 ± 2.43 bA | | 7.93 ± 2.25 aA | | 7.27 ± 0.91 aA | |
| SMA  (0.25-2 mm) | CK | 28.73 ± 0.51 cB | | 18.30 ± 0.26 aA | | 12.03 ± 0.21 aA | | 5.73 ± 0.47 aA | | 5.13 ± 0.57 bA | |  | | 52.90 ± 4.13 bA | | 14.07 ± 3.66 bA | | 6.27 ± 0.61 bB | | 5.87 ± 1.59 bcB | |
|  | CE | 34.63 ± 1.47 bA | | 16.83 ± 0.40 abB | | 12.03 ± 0.40 aB | | 6.67 ± 0.23 aA | | 6.33 ± 0.21 aA | |  | | 42.53 ± 1.29 aA | | 22.93 ± 3.20 aA | | 4.90 ± 1.14 aB | | 8.00 ± 1.15 aA | |
|  | WA | 38.63 ± 0.76 aB | | 15.80 ± 0.70 bB | | 9.20 ± 0.20 bB | | 5.90 ± 0.26 aAB | | 6.87 ± 0.21 aA | |  | | 64.63 ± 2.28 aA | | 10.60 ± 2.75 bAB | | 5.50 ± 1.25 abA | | 5.43 ± 1.65 abA | |
|  | CW | 30.57 ± 1.55 cA | | 18.07 ± 0.95 aA | | 12.33 ± 0.31 aB | | 7.17 ± 1.14 aA | | 5.90 ± 0.75 abA | |  | | 43.70 ± 1.11 cA | | 15.17 ± 3.29 abA | | 14.47 ± 6.26 cA | | 9.17 ± 1.60 cA | |
| MI  (< 0.25 mm) | CK | 32.47 ± 1.00 aA | | 18.40 ± 0.95 aA | | 11.43 ± 0.67 bA | | 5.77 ± 0.40 abA | | 4.97 ± 0.40 bB | |  | | 39.73 ± 5.87 bB | | 17.73 ± 4.88 abA | | 9.77 ± 1.21 aA | | 4.67 ± 1.46 aB | |
|  | CE | 28.90 ± 1.10 bB | | 19.77 ± 0.55 aA | | 12.70 ± 0.10 aA | | 5.97 ± 0.29 aA | | 5.77 ± 0.64 abA | |  | | 45.83 ± 3.85 aA | | 11.93 ± 0.76 aA | | 8.57 ± 0.81 aA | | 2.73 ± 0.15 aB | |
|  | WA | 33.20 ± 0.82 aC | | 18.20 ± 0.62 aA | | 11.40 ± 0.62 bA | | 5.70 ± 0.25 bB | | 5.70 ± 0.10 abB | |  | | 62.90 ± 2.15 aA | | 9.13 ± 0.75 bB | | 6.53 ± 1.29 aA | | 2.23 ± 0.51 aB | |
|  | CW | 29.73 ± 0.45 bA | | 19.43 ± 0.76 aA | | 12.27 ± 0.12 aB | | 6.27 ± 0.25 aA | | 6.27 ± 0.21 aA | |  | | 46.67 ± 2.27 cA | | 13.93 ± 1.50 aA | | 13.13 ± 1.44 bA | | 2.50 ± 0.56 bB | |
| *Factor (Df)* | | *F* | *p* | *F* | *p* | *F* | *p* | *F* | *p* | *F* | *p* |  | | *F* | *p* | *F* | *p* | *F* | *p* | *F* | *p* |
| *Aggregates (2)* | | *0.93* | *0.446* | *22.37* | ***** | *1.04* | *0.409* | *2.03* | *0.213* | *1.67* | *0.265* |  | | *0.65* | *0.556* | *3.63* | *0.093* | *6.83* | *** | *10.89* | *** |
| *Treatments (3)* | | *5.01* | *** | *1.83* | *0.243* | *5.82* | *** | *1.11* | *0.416* | *5.45* | *** |  | | *6.55* | *** | *2.52* | *0.154* | *8.99* | *** | *1.04* | *0.442* |
| *Aggregates × Treatments (6)* | | *21.30* | ***** | *3.93* | **** | *22.64* | ***** | *2.84* | *** | *2.85* | *** |  | | *5.27* | ***** | *2.77* | *** | *1.60* | *0.191* | *4.21* | **** |

CK, ambient control; CE, CO_2_ enrichment; WA, canopy warming; CW, CO_2_ enrichment plus canopy warming; LMA, large macro-aggregates (> 2 mm); SMA, small macro-aggregates (0.25–2 mm); MI, micro-aggregates (< 0.25 mm). Different letters indicate statistical differences (*p* < 0.05) among different treatments in each aggregate size (lowercase letters) and different aggregate sizes in each treatment (uppercase letters). The asterisks ‘***’, ‘**’and ‘*’ indicate significant levels with at *p* < 0.001, *p* < 0.01 and *p* < 0.05, respectively

**Table S8** Topological properties of bacterial and fungal co-occurrence network in soil aggregate sizes under long-term climate change.

| Network | Bacteria | | | | | | | Fungi | | | | | | |
| --- | --- | --- | --- | --- | --- | --- | --- | --- | --- | --- | --- | --- | --- | --- |
|  | CK | CE | WA | CW | LMA | SMA | MI | CK | CE | WA | CW | LMA | SMA | MI |
| Nodes | 678 | 559 | 524 | 618 | 396 | 353 | 394 | 149 | 259 | 163 | 198 | 129 | 121 | 170 |
| Edges | 918 | 751 | 527 | 869 | 563 | 370 | 564 | 128 | 314 | 171 | 198 | 188 | 198 | 264 |
| Average degrees | 3.544 | 2.687 | 2.011 | 3.224 | 2.834 | 2.096 | 2.863 | 1.718 | 2.425 | 2.098 | 2.000 | 2.915 | 3.273 | 3.106 |
| Average path length | 10.427 | 7.574 | 7.794 | 8.529 | 6.704 | 7.942 | 5.600 | 4.475 | 7.336 | 6.216 | 8.033 | 5.611 | 4.073 | 4.954 |
| Negative edges | 43.03% | 47.14% | 57.50% | 45.68% | 49.92% | 55.41% | 53.01% | 83.59% | 67.20% | 81.29% | 72.22% | 62.23% | 63.64% | 57.95% |
| Positive edges | 56.97% | 52.86% | 42.50% | 54.32% | 50.98% | 44.59% | 46.99% | 16.41% | 32.80% | 18.71% | 27.78% | 37.77% | 36.36% | 42.05% |

CK, ambient control; CE, CO_2_ enrichment; WA, canopy warming; CW, CO_2_ enrichment plus canopy warming; LMA, large macro-aggregates (> 2 mm); SMA, small macro-aggregates (0.25–2 mm); MI, micro-aggregates (< 0.25 mm). Different letters indicate statistical differences (*p* < 0.05) among treatments or aggregate sizes

**Fig. S1** The contribution of microbial necromass (a), bacterial necromass (b), and fungal necromass (c) in soil organic carbon (SOC) in aggregate fractions. CK, ambient control; CE, CO_2_ enrichment; WA, canopy warming; CW, CO_2_ enrichment plus canopy warming. LMA, large macro-aggregates (> 2 mm); SMA, small macro-aggregates (0.25–2 mm); MI, micro-aggregates (< 0.25 mm). Different letters indicate statistical differences among different treatments in each aggregate.

**Fig. S2** Soil bacterial and fungal compositions at the phylum level in soil aggregates under different treatments. CK, ambient control; CE, CO_2_ enrichment; WA, canopy warming; CW, CO_2_ enrichment plus canopy warming. LMA, large macro-aggregates (> 2 mm); SMA, small macro-aggregates (0.25–2 mm); MI, micro-aggregates (< 0.25 mm).

**Fig. S3** Microbial functional groups in soil aggregates under climate change. Soil bacterial function profiles predicted based on the FAPROTAX database (a); soil fungal guilds predicted based on FUNGuild (b). CK, ambient control; CE, CO_2_ enrichment; WA, canopy warming; CW, CO_2_ enrichment plus canopy warming. LMA, large macro-aggregates (> 2 mm); SMA, small macro-aggregates (0.25–2 mm); MI, micro-aggregates (< 0.25 mm).

**Fig. S4** Bacterial functional groups in soil aggregates under long-term climate change. Bacterial functional groups were predicted by BugBase tool. CK, ambient control; CE, CO_2_ enrichment; WA, canopy warming; CW, CO_2_ enrichment plus canopy warming. LMA, large macro-aggregates (> 2 mm); SMA, small macro-aggregates (0.25–2 mm); MI, micro-aggregates (< 0.25 mm). Different uppercase letters represent statistical differences (*P*<0.05) among treatments in each aggregate fraction.

**Fig. S5** Bacterial metabolic pathways and functions in soil aggregates under long-term climate change. Bacterial pathways and functions were categorized into Kyoto Encyclopedia of Genes and Genomes (KEGG) database with the PICRUSt2 algorithm. CK, ambient control; CE, CO_2_ enrichment; WA, canopy warming; CW, CO_2_ enrichment plus canopy warming. LMA, large macro-aggregates (> 2 mm); SMA, small macro-aggregates (0.25–2 mm); MI, micro-aggregates (< 0.25 mm).

**Fig. S6** Soil enzyme activities in soil aggregates under long-term climate change. Different letters indicate statistical differences among different treatments (lowercase letters) and different aggregate fractions (uppercase letters). BG, *β*-glucosidase; CB, *β*-cellobiosidase; XYL, β-xylosidase; NAG, N-acetyl-*β*-glucosaminidase; LAP, leucine aminopeptidase; PHOS, neutral phosphatase. The values are two-way ANOVA analyses on effects of the aggregate fractions (A), climate change treatments (T) and their interactions (A × T). The asterisks ‘***’, ‘**’and ‘*’ indicate significant levels with at *p* < 0.001, *p* < 0.01 and *p* < 0.05, respectively. CK, ambient control; CE, CO_2_ enrichment; WA, canopy warming; CW, CO_2_ enrichment plus canopy warming. LMA, large macro-aggregates (> 2 mm); SMA, small macro-aggregates (0.25–2 mm); MI, micro-aggregates (< 0.25 mm). The values in parentheses are the effects of soil aggregate size (A), climate change treatments (T) and their interactions (A × T).

**Fig. S7** Correlations between bacterial (a) and fungal (b) communities and soil environmental factors, enzyme activities, and functional genes in soil aggregates. Environmental factors include soil pH, soil organic carbon (SOC), total nitrogen (TN), and total phosphorus (TP). Microbial genes include bacterial gene abundance (Bac) and fungal gene abundance (Fun). Soil enzyme activities include *β*-glucosidase (BG), *β*-cellobiosidase (CB), β-xylosidase (XYL), N-acetyl-*β*-glucosaminidase (NAG), leucine aminopeptidase (LAP), neutral phosphatase (PHOS). LMA, large macro-aggregates (> 2 mm); SMA, small macro-aggregates (0.25–2 mm); MI, micro-aggregates (< 0.25 mm).

**Fig. S8** Correlation of soil properties, enzymatic stoichiometry, microbial diversity and gene abundance in soil aggregates. SOC, soil organic carbon; TN, Total nitrogen; TP, total phosphorus; B_Shannon, bacterial Shannon‒Wiener index; B_ACE, bacterial ACE; B_Chao1, bacterial Chao1; B_abundance, bacterial gene abundance; F_Shannon, fungal Shannon‒Wiener index; F_ACE, fungal ACE; F_Chao1, fungal Chao1; F_abundance, fungal gene abundance; C-acq, C-acquiring enzyme activity; N-acq, N-acquiring enzyme activity; P-acq, P-acquiring enzyme activity. LMA, large macro-aggregates (> 2 mm); SMA, small macro-aggregates (0.25–2 mm); MI, micro-aggregates (< 0.25 mm). Asterisks denote significant correlation: **P* < 0.05; ***P* < 0.01; ****P* < 0.001.

**Fig. S9** Relationships between the microbial necromass C with soil pH (a, b, c) and SOC (d, e, f) in soil aggregates. The shaded bands show a 95% pointwise confidence interval of the linear regression model. SOC, soil organic carbon; LMA, large macro-aggregates (> 2 mm); SMA, small macro-aggregates (0.25–2 mm); MI, micro-aggregates (< 0.25 mm).
